# Ultrasonography Assessment of Neck Anatomy for Prediction of Difficult Mask Ventilation in Obese Patients: A Prospective Observational Study †

**DOI:** 10.3390/diagnostics15131615

**Published:** 2025-06-25

**Authors:** Ignatius Wong Hsun-Hong, Azarinah Izaham, Syarifah Noor Nazihah Sayed Masri, Muhammad Maaya, Siti Nidzwani Mohamad Mahdi, Khazrul Nizar Abd Kader, Maryam Budiman, Yazmin Yaacob

**Affiliations:** 1 Department of Anaesthesiology and Intensive Care, Faculty of Medicine, Universiti Kebangsaan Malaysia, Jalan Yaacob Latif, Bandar Tun Razak, Cheras, Kuala Lumpur 56000, Malaysia; ignatiusw88@gmail.com (I.W.H.-H.); syarifahnoornazihah@gmail.com (S.N.N.S.M.); muhammad@hctm.ukm.edu.my (M.M.); nidzwani@hctm.ukm.edu.my (S.N.M.M.); kai_naza@yahoo.co.uk (K.N.A.K.); maryam.budiman@hctm.ukm.edu.my (M.B.); 2 Department of Radiology, Faculty of Medicine, Universiti Kebangsaan Malaysia, Jalan Yaacob Latif, Bandar Tun Razak, Cheras, Kuala Lumpur 56000, Malaysia; minyaacob@yahoo.com

**Keywords:** airway management, ultrasonography neck, difficult mask ventilation

## Abstract

**Background**: Effective mask ventilation is a very important aspect of ensuring adequate oxygenation and ventilation. However, predicting difficult mask ventilation (DMV) using bedside clinical tests remains challenging due to poor sensitivity. Our objective was to determine the correlation between the preoperative ultrasonography of anterior neck anatomy and difficult mask ventilation in different obesity classes. **Methods**: A prospective, observational study enrolled 90 adult obese patients undergoing general anesthesia from December 2020 to November 2021 (30 patients for each class of obesity). Ultrasonography measurements were recorded for the distance of skin (DS) to hyoid bone (DSHB), epiglottis (DSEM), the anterior commissure of the vocal cords (DSAC), thyroid isthmus (DSTI), and trachea at jugular notch (DSTJ). The difficulty of bag mask ventilation was graded using the Han scale. The Kendall Tau correlation coefficient was used to correlate the different ultrasonography parameters to DMV. Receiver-operating characteristic (ROC) curves were used to determine the sensitivity and specificity of the measured ultrasonography distances, and the Youden index was used to calculate the optimal cut-off values. **Results**: Results revealed twenty patients (22.2%) were categorized as having difficult mask ventilation. There was a statistically significant increase (*p* = 0.011) in the number of patients with Mallampati II in class III obesity compared to class I obesity. DSHB showed a statistically significant and strong correlation with difficult mask ventilation in patients with class II (*p* = 0.002, r = 0.464) and class III obesity (*p* = 0.002, r = 0.475). A DSHB cut-off value of 1.35 cm has a sensitivity of 83.3% and specificity of 78.8% for class III obesity. Similarly, a DSTJ cut-off value of 1.13 cm has a sensitivity of 83.3% and specificity of 66.7% for class III obesity. **Conclusions**: Notably, DSHB was the most specific parameter and equally as sensitive as DSTJ in predicting difficult mask ventilation in morbidly obese patients.

## 1. Introduction

Clinical guidelines have provided indices to predict the risk of difficult mask ventilation (DMV), though the incidence still varies between 0.08 and 15% [1,2,3]. A study by Cattano et al. reported that the incidence of difficult mask ventilation was 8.9% in general surgery patients [1]. Effective mask ventilation is important in ensuring adequate oxygenation and ventilation [4]. The American Society of Anesthesiologists (ASA) Guidelines for Management of the Difficult Airway defines difficult facemask ventilation as the situation in which “it is not possible for the anesthesiologist to provide adequate face mask ventilation due to one or more of the following problems: inadequate mask seal, excessive gas leak, or excessive resistance to ingress or egress of gas” [5]. Han et al. have suggested a scale to grade difficult mask ventilation [6] (Appendix A).

In difficult mask ventilation, critical hypoxemia can rapidly lead to life-threatening complications including myocardial and cerebral infarction. The risk is heightened in obese patients due to altered external airway anatomy, increased parapharyngeal fat and restrictive pulmonary changes that reduce oxygen reserves and shorten safe apnea time. Any tool predicting DMV can enhance airway management safety as an adjunct to conventional assessments. Ultrasonography offers quick, relatively easy, and accurate diagnostic information with clinical relevance [7,8,9].

A study by Alessandri et al. used ultrasonography of the airway to predict difficult mask ventilation in Ear, Nose, Throat patients [10]. Alessandri et al. measured several parameters and assessed the correlation of neck parameters to actual difficult mask ventilation in the operating theater. In their study, patients with abnormal airways were excluded, as the intention was to aid DMV detection.

The obese population has shown that anatomical and physiological changes in obesity can lead to rapid oxygen desaturation [11]. Hence, it would be important to determine whether obese patients can be bag-mask ventilated during the apneic period. The objective of this study was to determine the correlation of preoperative ultrasonography of neck anatomy to difficult mask ventilation in different classes of obesity.

## 2. Materials and Methods

This prospective, observational clinical study was conducted at Universiti Kebangsaan Malaysia Medical Centre (UKMMC). This study commenced after obtaining institutional ethics committee approval (Research project code: JEP-2020-561) from December 2020 to November 2021. Authors followed the Strengthening the Reporting of Observational Studies in Epidemiology (STROBE) guidelines when writing and reporting this study (File S1). The inclusion criteria were obese patients aged over 18 years with American Society of Anesthesiologists (ASA) classification I-III who were scheduled to undergo general anesthesia. Excluded were patients with head and neck trauma, tumor or previous surgery, jaw protrusion, beard, pregnancy, kyphoscoliosis, gastroesophageal reflux disease, and Mallampati III and IV classification.

A patient information sheet was provided, and written informed consent was obtained. A total of 90 patients were recruited and categorized into three different obesity classes as defined in the Malaysian Clinical Practice Guidelines on Management of Obesity (2004): class I obesity was defined as BMI 27.5 kg/m^2^ to 34.9 kg/m^2^, class II obesity as BMI 35 kg/m^2^ to 39.9 kg/m^2^, and class III obesity as BMI > 40 kg/m^2^ [12].

Patients were assessed by the primary investigator in the operating theater on the day of surgery. The primary investigator, who had undergone training under a consultant radiologist’s supervision, was the sole operator performing ultrasonography scans for recruited patients prior to general anesthesia. Before the ultrasonography scan, patients’ heads were positioned with the external auditory meatus aligned with the sternal notch—the same position maintained during anesthesia induction and mask ventilation.

**Figure 1:**
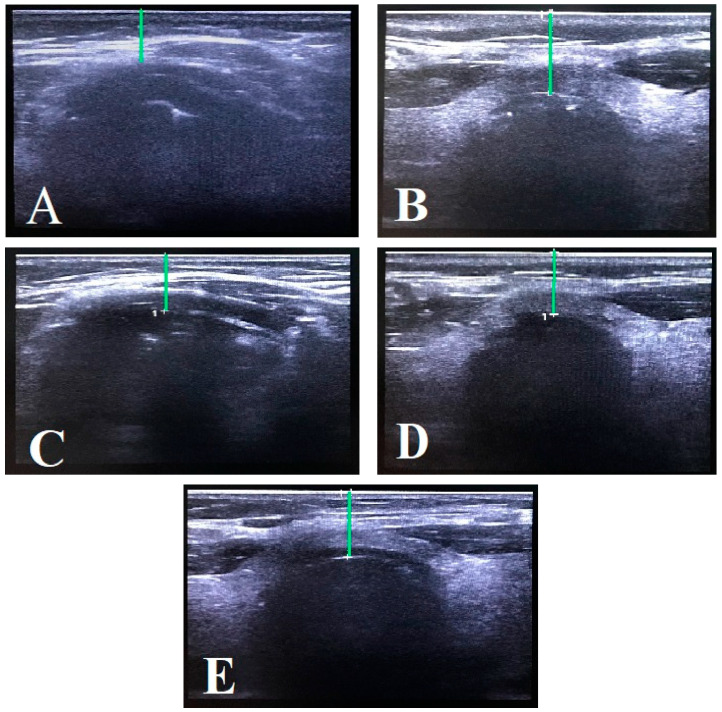
Ultrasonography distances: (**A**) DSHB. (**B**) DSEM. (**C**) DSAC. (**D**) DSTI. (**E**) DSTJ.

Anterior neck soft tissue thickness was measured using a portable ultrasound machine (Fujifilm SonoSite SII manufactured by FUJIFILM Sonosite, Inc., Bothell, WA, USA) with a linear transducer probe. Scans were performed on the anterior neck from cranial to caudal, with the probe placed in the transverse axis. The following parameters were measured and recorded: distance from skin to hyoid bone (DSHB), distance from skin to epiglottis (DSEM), distance from skin to anterior commissure of the vocal cords (DSAC), distance from skin to thyroid isthmus (DSTI), and distance from skin to trachea at the jugular notch level (DSTJ) in Figure 1.

In the operating room, all patients were positioned in the same elevated head-up position used during ultrasonography scans. Preoxygenation was performed with 100% oxygen for 3 min, increasing end-tidal oxygen to a minimum of 80 mmHg. Anesthesia was induced with intravenous propofol (2 mg/kg), fentanyl (2 μg/kg), and rocuronium (0.9 mg/kg). Gentle mask ventilation was performed using a clear disposable plastic mask with 100% oxygen by the investigator.

Successful bag mask ventilation was characterized by chest rise, capnography, tidal volume of at least 6 mL/kg, and minimal oxygen saturation of 95% for 1 min. Difficult mask ventilation (DMV) grades were recorded using the Han scale, with Grades 1 and 2 classified as easy mask ventilation and Grades 3 and 4 as difficult mask ventilation [6]. The ultrasonography measurements of neck anatomy and DMV grades were recorded and categorized according to obesity classes. In case of desaturation or inability to ventilate, rescue steps followed the Difficult Airway Society protocol [13]. Study drop-outs included patients who experienced discomfort and refused to continue or had surgery canceled.

### Statistical Analysis

The sample size was calculated using G*Power software (version 3.1.9.2). Based on the specific objective, the sample size calculation was to determine the estimated number of patients to obtain a positive correlation result; hence, in this study, it was based on the parameter DSHB, which was the most sensitive parameter on a previous study by Alessandri et al., in which r was 0.5 [10]. The power of this study was set at 80%, and α value was set at 0.05. The sample size needed, including 10% dropout rate, was 27 patients for each class of obesity. In this study, we managed to obtain 30 patients for each class of obesity. This sample size calculation was as follows:

The standard normal deviate for α = Z α = 1.960 at 0.05 alpha value,(1)

The standard normal deviate for β = Z β = 0.842 at 80% power study(2)

All data analyses were performed using SPSS 26.0 (Statistical Package for The Social Sciences) for Windows version 26.0 (IBM Corp, Armonk, NY, USA). Results were expressed as mean ± standard deviation, median (interquartile range), or frequency (percentage) as appropriate. Correlation analysis was performed using Kendall’s tau-b (τ_b_) correlation coefficient to correlate the different ultrasonography parameters to DMV. A *p* value of <0.05 will be considered as statistically significant. Further analysis using receiver-operating characteristic (ROC) curves was used to determine the sensitivity and specificity of the measured ultrasound distances to predict difficult mask ventilation. Optimal cutoff values were calculated using the Youden index (calculated as sensitivity + specificity − 1) [14].

## 3. Results

A total of 90 patients were included in this study. The demographic data, personal, and clinical characteristics are presented in Table 1. A statistically significant increase (*p* = 0.011) was observed in the number of patients with Mallampati II classification in class III obesity compared to class I obesity. Out of the 90 patients, 20 patients (22%) were classified as having difficult mask ventilation (Han Grade 3 and 4), as shown in Table 2.

Table 3 summarizes, among various ultrasonography measurements, that the distance from skin to hyoid bone (DSHB) has a statistically significant and strong correlation with difficult mask ventilation (DMV), but only in patients with class II and III obesity. Other ultrasonographic parameters that were measured did not show statistical significance in predicting difficult mask ventilation.

**Figure 2:**
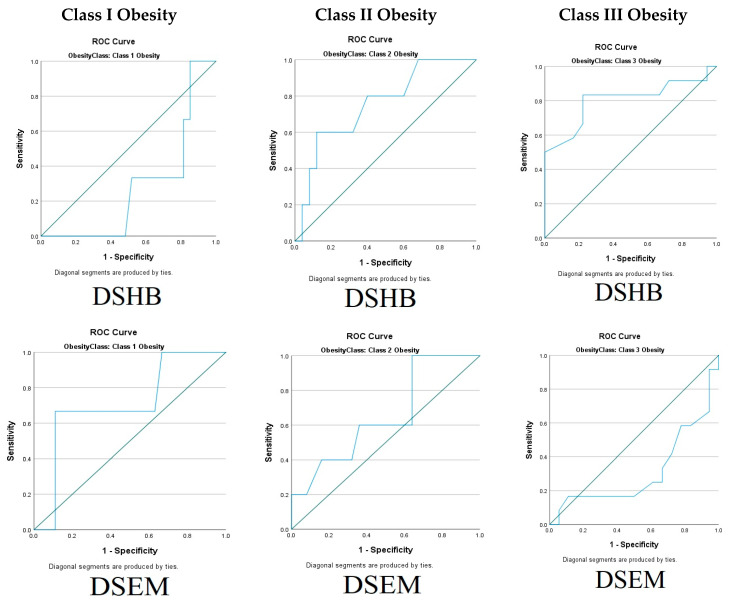

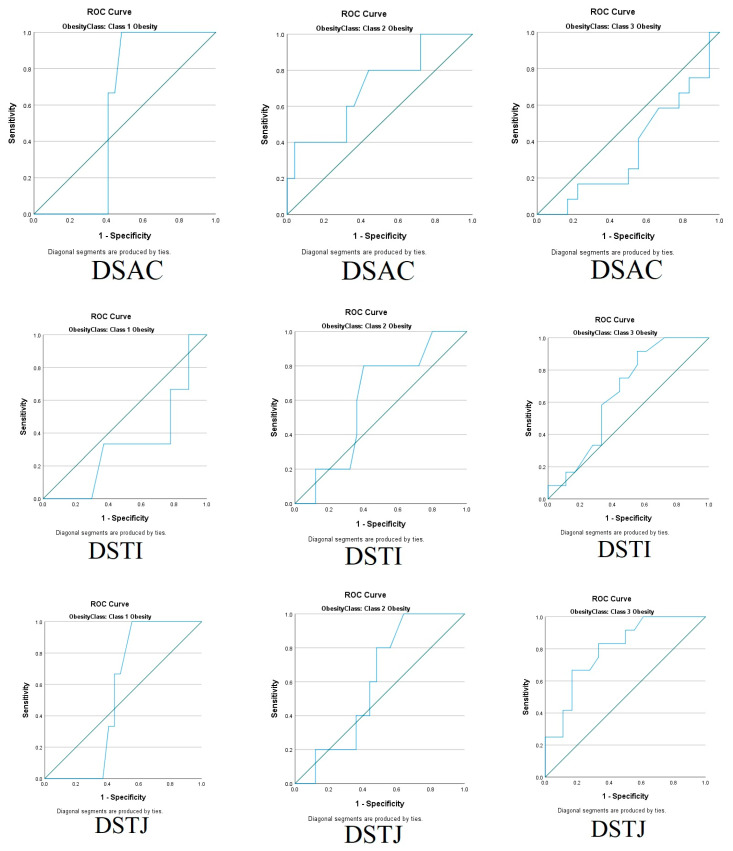
Receiver operating characteristic (ROC) curve analyses of the 5 ultrasonographic measurements and DMV. Distance of skin (DS) to hyoid bone (DSHB), epiglottis (DSEM), anterior commissure of the vocal cords (DSAC), thyroid isthmus (DSTI), and trachea at jugular notch (DSTJ). *p* < 0.05. Class I for DSHB: sensitivity = 33.0%; specificity = 22.0%. Class I for DSEM: sensitivity = 66.7%; specificity = 88.0%. Class I for DSAC: sensitivity = 100%; specificity = 51.9%. Class I for DSTI: sensitivity = 33.0%; specificity = 22.0%. Class I for DSTJ: sensitivity = 100%; specificity = 44.4%. Class II for DSHB: sensitivity = 60.0%, specificity = 88.0%. Class II for DSEM: sensitivity =100%; specificity = 36.0%. Class II for DSAC: sensitivity = 80%; specificity = 56.0%. Class II for DSTI: sensitivity = 80.0%; specificity = 60.0%. Class II for DSTJ: sensitivity = 100%; specificity = 36.0%. **Class III for DSHB: sensitivity = 83.3%; specificity = 77.8%**. Class III for DSEM: sensitivity = 25%; specificity = 33.3%. Class III for DSAC: sensitivity = 16.7%; specificity = 50.0%. Class III for DSTI: sensitivity =75.0%; specificity = 55.6%. **Class III for DSTJ: sensitivity = 83.3%; specificity = 66.7%**.

The correlation between ultrasonography distances and Han grade for mask ventilation is shown by comparison of the area under the ROC curves in Figure 2. The correlation showed that the best predictors of DMV scale Han Grade 3 and 4 were DSHB at 1.35 cm using the Youden index with a sensitivity of 83.3% and specificity of 78.8% [Area Under the Curve (AUC) 0.803; 95% confidence interval 0.621 to 0.985] and DSTJ at 1.13 cm with a sensitivity of 83.3% and specificity of 66.7% [Area Under the Curve (AUC) 0.796; 95% confidence interval 0.637 to 0.955] for class III obesity. For obesity classes I and II, ultrasonography distances were unable to differentiate between easy and difficult mask ventilation statistically.

In contrast, for obesity classes I and II, the ultrasonography distances were unable to statistically differentiate between easy and difficult mask ventilation. This finding suggests that the predictive value of these ultrasonographic measurements may be more pronounced in patients with more severe obesity.

## 4. Discussion

Airway assessments using ultrasonography have recently become increasingly popular as more studies seek a more reliable screening tool for difficult airways (difficult mask ventilation and difficult intubation). This is because bedside clinical parameters have been shown to have limited predictive value and poor diagnostic accuracy. A recent Cochrane Systematic Review in 2018 concluded that future research is needed to develop tests with higher sensitivities to better screen for difficult mask ventilation and difficult intubation [15].

The advantages of ultrasonography include that it can be used at bedside, it is fast, it is radiation-free, and it provides real-time assessment. The entire scanning of the five parameters using ultrasonography can be completed within five minutes. Moreover, studies have shown that ultrasonography, when performed correctly, has an accuracy matching airway magnetic resonance imaging (MRI) [16]. Computer tomography (CT) and magnetic resonance imaging (MRI) are good modalities for measuring neck soft tissue thickness, but they are expensive, unavailable in the operating theater, and time consuming [16,17].

The results of our study showed that 22.2% of patients experienced difficult mask ventilation. The DSHB demonstrated a strong correlation with DMV in class II and III obesity. As for class III obesity, DSHB measurements above 1.35 cm predicted DMV with 83.3% sensitivity and 78.8% specificity. However, the DSTJ above 1.13 cm showed similar sensitivity (83.3%) but lower specificity (66.7%). So, we conclude that DSHB is the most reliable ultrasound parameter for predicting difficult mask ventilation in morbidly obese patients.

In our study, we found an increase in the number of patients with Mallampati II in class III obesity compared to class I obesity. A study by Menon et al. on 323 patients demonstrated that an increased Body Mass Index (BMI) positively correlates with an increase in Mallampati score [18]. Mallampati III and IV are identified as independent predictors of difficult mask ventilation [19]. However, these patient groups were excluded from this study to avoid potential bias.

There was no statistically significant difference in thyromental distance (TMD) and the inter-incisor gap between different obesity classes in this study. Shailaja et al., who conducted a study on 200 patients comparing intubation ease between lean and obese patients, also showed no statistical difference in TMD (*p* = 0.597) and the inter-incisor gap (*p* = 0.081) between patient groups [20].

Our study found that the percentage of difficult mask ventilation was 22.2% of the total sample, with 3.3% in class I obesity, 5.6% in class II obesity, and 13.3% in class III obesity. This aligns with other studies involving difficult mask ventilation in obese patients, which reported a range of 2.9–38.9% for difficult mask ventilation [1,21,22]. Yildiz et al. and Moon et al. demonstrated that increasing BMI is significantly associated with difficult mask ventilation (DMV), which is consistent with our findings [21,22]. We found that Deep Soft Tissue Height at Base (DSHB) is statistically significant and strongly correlates with difficult mask ventilation (DMV) in obesity classes II and III [15]. Using the Youden index, we identified a DSHB value of 1.35 cm with high sensitivity and specificity in obesity class III. At the time of this research, there were not many other studies conducted with the same parameters to predict difficult mask ventilation. Though the hyoid bone is known to be responsible for airway collapsibility, to date, there is no study correlating with difficult DMV.

A similar study by Alessandri et al., using the same five parameters on patients undergoing elective ear, nose, and throat surgeries, also exhibited a statistically positive association between increased thickness of the anterior neck soft tissues to the incidence of DMV, with DSHB exhibiting the highest correlation [10]. It was postulated because the hyoid acts as the fulcrum of the upper airway as it is connected to the tongue by the genioglossus muscle and to the larynx through the hyoepiglottic and thyrohyoid membranes.

However, as our study specifically focused on the obese population, we observed that skin-to-structure distances varied significantly across different obesity classifications. We only found the strongest predictive relationship between ultrasound measurements, and difficult mask ventilation was found exclusively in class III obesity. This finding suggests that the clinical utility of these ultrasound parameters may vary in the different classes of obesity.

Comparing the results from this study to bedside clinical test sensitivity and specificity in predicting difficult mask ventilation, data from a systematic review by Roth et al. showed that bedside clinical tests have low sensitivity in predicting difficult mask ventilation. Recent studies published this year studied different ultrasonographic parameters such as tongue base thickness and distance between lingual arteries [15].

Lin et al. conducted a study on 28 patients of the general population and found that the sensitivity and specificity of tongue base thickness were 50% and 87%, whereas the sensitivity and specificity of the distance between lingual arteries had a sensitivity of 88.9% and specificity of 65.2% [23]. Padhy et al. conducted a study on 127 patients with obstructive sleep apnea and found that the sensitivity and specificity for tongue base thickness were 83.0% and 81.2%, while the sensitivity and specificity for the distance between lingual arteries are 90.6% and 82.4%, respectively [24].

Comparing that to our study, DSHB has a sensitivity of 83.3% and specificity of 78.8%, whereas Deep Soft Tissue at Junction (DSTJ) has a sensitivity of 83.3% and specificity of 66.7%. All these parameters show that ultrasonographic parameters have a higher sensitivity than bedside clinical tests and can be used as a groundwork for further studies with a larger sample size in the future.

While more studies are required on these ultrasonography parameters to establish a specific scanning protocol, we can deduce that ultrasonography of the neck parameters gives a reasonably high sensitivity and specificity and can be considered as an adjunct to bedside clinical parameters in predicting difficult mask ventilation.

## 5. Limitations

The limitations of our study are that it was a pilot study carried out in a single center only. Therefore, our findings may not be applicable to other areas or the larger population. Ultrasonography also requires specific skill and experience in order to achieve reliable and consistent ultrasonography scan results. However, ultrasonography of the neck anatomy has a short learning curve as the location of the upper airway is fixed and does not require much adjustment once the location is identified and the anatomy is consistent among patients, even in the obese [25]. Since obese patients with difficult mask ventilation have a high Mallampati classification of III and IV, we categorized patients by BMI range to determine which specific obesity classification demonstrates the strongest correlation with DMV when assessed using ultrasound measurements.

Additionally, we are aware that sole operators performing ultrasonography would introduce bias. To address this limitation, the principal investigator underwent specialized training with a consultant radiologist to ensure proper technique and interpretation. We acknowledge that, while neck ultrasound is not yet common practice among anesthesiologists, initial training with radiology experts is essential to establish competency. Following this training period, we believed the technique could be incorporated into routine anesthetic practice. This approach to specialist training followed by skill dissemination aligns with the broader trend of anesthesiologists expanding their ultrasound capabilities beyond traditional applications.

## 6. Conclusions

Based on the results of our study, we found that DSHB was the most sensitive and specific parameter compared to other ultrasonographic parameters in predicting difficult mask ventilation in morbidly obese patients. It suggests that the predictive value of these ultrasonographic measurements may be more pronounced in patients with more severe obesity. Thus, this approach provides more nuanced clinical guidance by identifying which obesity subgroups may benefit most from an ultrasound-based airway assessment. Recommending a future study in patients with obesity on a different population is warranted.

## Figures and Tables

**Table 1:** Patients’ demographic data and clinical parameters.

	Obesity			*p* Value
	Class I (*n* = 30)	Class II (*n* = 30)	Class III (*n* = 30)	
Age	55.80 ± 14.5	50.80 ± 16.50	48.10 ± 16.60	0.169
Gender				0.873
Male	12 (40.00)	13 (43.30)	14 (46.70)	
Female	18 (60.00)	17 (56.70)	16 (53.30)	
ASA physical status				0.121
I	4 (13.30)	8 (26.70)	2 (6.70)	
II	15 (50.00)	16 (53.30)	14 (46.70)	
III	11 (36.70)	6 (20.00)	14 (46.70)	
Mallampati score				
I	7 (23.30)	1 (3.30)	0 (0.00)	0.005 *
II	23 (76.70)	29 (96.70)	30 (100.00)	
Thyromental distance				
>6 cm	22 (73.30)	26 (86.70)	19 (63.30)	0.115
<6 cm	8 (26.70)	4 (13.30)	11 (36.70)	
Mouth opening				0.540
(inter-incisor gap)				
>3 cm	30 (100.00)	28 (93.30)	28 (93.30)	
<3 cm	0 (0.00)	2 (6.70)	2 (6.70)	
Ultrasound distances				
DSHB	1.05 (0.22)	1.06 (0.11)	1.30 (0.16)	0.045 *
DSEM	1.43 (0.24)	1.32 (0.24)	1.64 (0.11)	0.038 *
DSAC	1.08 (0.28)	0.99 (0.16)	1.26 (0.15)	0.575
DSTI	0.77 (0.20)	0.80 (0.08)	0.79 (0.16)	0.175
DSTJ	0.99 (0.19)	0.94 (0.18)	1.19 (0.13)	0.207

Numerical data are expressed as mean (SD) and number (%). *p* value < 0.05. Post hoc *p* value for Mallampati II: CI vs. CII = 0.052 *, CI vs. CIII = 0.011 *, and CII vs. CIII = 1.000. Post hoc *p* value for DSHB: CI vs. CII = 0.580, CI vs. CIII = 0.158, and CII vs. CIII = 0.008 *. Post hoc *p* value for DSEM: CI vs. CII = 0.441, C1 vs. CIII = 0.095, and CII vs. CIII = 0.038 *.

**Table 2:** Difficult mask ventilation for Han grades and different classes of obesity.

		Obesity	
	Class I (*n* = 30)	Class II (*n* = 30)	Class III (*n* = 30)
Han Grade 1	9 (30.0)	9 (30.0)	-
Han Grade 2	18 (60.0)	16 (53.3)	18 (60.0)
Han Grade 3	3 (10.0)	5 (16.7)	10 (33.3)
Han Grade 4	-	-	2 (6.7)

Numerical data are expressed as number (%).

**Table 3:** The ultrasonography distances graded for DMV (Han Grade 3 and 4) and the correlation of ultrasonography distances with DMV using Kendall’s tau-b (τb) correlation coefficient.

	Obesity					
	Class I (*n* = 30)		Class II (*n* = 30)		Class III (*n* = 30)	
	Easy (*n* = 27)	Difficult (*n* = 3)	Easy (*n* = 25)	Difficult (*n* = 5)	Easy (*n* = 18)	Difficult (*n* = 12)
DSHB	1.06 ± 0.220	0.92 ± 0.130	1.05 ± 0.110	1.15 ± 0.130	1.26 ± 0.18	1.36 ± 0.11
	*p* = 0.934, r 0.012		* *p* **= 0.002, r 0.464**		* *p* **= 0.002, r 0.475**	
DSEM	1.42 ± 0.240	1.56 ± 0.210	1.29 ± 0.180	1.49 ± 0.430	1.67 ± 0.08	1.60 ± 1.13
	*p* = 0.375, r 0.133		*p* = 0.082, r 0.264		*p* = 0.196, r 0.197	
DSAC	1.08 ± 0.240	1.07 ± 0.030	0.98 ± 0.140	1.12 ± 0.210	1.29 ± 0.18	1.22 ± 0.11
	*p* = 0.885, r 0.022		*p* = 0.106, r 0.244		*p* = 0.436, r 0.118	
DSTI	0.79 ± 0.210	0.67 ± 0.140	0.80 ± 0.090	0.81 ± 0.050	0.76 ± 0.13	0.84 ± 0.19
	*p* = 0.665, r 0.065		*p* = 0.594, r 0.082		*p* = 0.119, r 0.237	
DSTJ	0.99 + 0.200	0.99 ± 0.020	0.94 ± 0.200	0.95 ± 0.100	1.13 ± 0.11	1.27 ± 0.12
	*p* = 0.984, r 0.003		*p* = 0.473, r 0.109		*p* = 0.101, r 0.248	

Numerical data are expressed as mean (SD) and number (%). * *p* value < 0.05.

## Data Availability

The authors can provide access to the dataset that was generated and/or analyzed during the present study upon request, subject to reasonable conditions.

## Supplementary Materials

The following supporting information can be downloaded at https://www.mdpi.com/article/10.3390/diagnostics15131615/s1, File S1: The STROBE Statement Checklist.

## Abbreviations

The following abbreviations are used in this manuscript:

ASA	American Society of Anesthesiologists
AUC	Area Under the Curve
CT	Computer tomography
DMV	Difficult mask ventilation
DS	Distance of skin
DSHB	Distance from skin to hyoid bone
DSEM	Distance from skin to epiglottis
DSAC	Distance from skin to anterior commissure of the vocal cords
DSTI	Distance from skin to thyroid isthmus
DSTJ	Distance from skin to trachea at the jugular notch level
DSTJ	Deep Soft Tissue at Junction
ROC	Receiver-operating characteristic
MRI	Magnetic resonance imaging
TMD	Thyromental distance
UKMMC	Universiti Kebangsaan Malaysia Medical Centre

## Appendix A

**Table A1:** Han’s scale grade of difficult mask ventilation.

Classification	Description
Grade 1	Ventilated by mask
Grade 2	Ventilated by mask with oral airway/adjuvant
Grade 3	Difficult mask ventilation (inadequate, unstable, or requiring two practitioners)
Grade 4	Unable to mask-ventilate
